# Some new inequalities for continuous fusion frames and fusion pairs

**DOI:** 10.1186/s40064-016-3284-0

**Published:** 2016-09-19

**Authors:** Wei Zhang, Yun-Zhang Li

**Affiliations:** College of Applied Sciences, Beijing University of Technology, Beijing, 100124 People’s Republic of China

**Keywords:** Continuous fusion frame, Parseval continuous fusion frame, Fusion pair, 42C15, 42C40

## Abstract

This paper addresses continuous fusion frames and fusion pairs which are extensions of discrete fusion frames and continuous frames. The study of equalities and inequalities for various frames has seen great achievements. In this paper, using operator methods we establish some new inequalities for continuous fusion frames and fusion pairs. Our results extend and improve ones obtained by Balan, Casazza and Găvruţa.

## Background

The notion of frame in a general Hilbert space was first introduced by Duffin and Schaeffer in 1952 to study nonharmonic Fourier series (Duffin and Schaeffer 1952). However, the frame theory had not interested many researchers until Daubechies, Crossman and Meyer published their ground breaking work in Daubechies et al. (1986). In recent years, the study of frame theory has seen great achievements, and frames are widely used in signal processing, quantum measurements, image processing, coding and communication and some other fields (Balan et al. 2007; Bownik et al. 2015; Casazza 2000; Christensen 2003; Leng and Han 2013; Li and Sun 2008; Li and Zhu 2012; Li et al. 2015; Rahimi et al. 2006; Strohmer and Heath 2003). The study of fusion frames dates back to Casazza and Kutyniok (2004) by Casazza et al. (2008) by Casazza, Kutyniok and Li, which can be used in distributed processing. As Casazza, Kutyniok and Li pointed out in Casazza et al. (2008), in applications, one is often overwhelmed by a deluge of data assigned to one single frame system, which becomes simply too large to be handled numerically. In these cases it would be highly beneficial to split a large frame system into a set of (overlapping) much smaller systems, and to process locally within each sub-system effectively. The notion of continuous fusion frame is a generalization of the above discrete fusion frames, It was first introduced by Faroughi and Ahmadi in Faroughi and Ahmadi (2008). This paper focuses on some inequalities for continuous fusion frames and fusion pairs.

In applications such as speech recognition, it was a longstanding conjecture by many engineers that a signal can be reconstructed without information about the phase. In 2006, Balan, Casazza and Edidin verified this conjecture (Balan et al. 2006). While working on efficient algorithms for signal reconstruction, Balan, Casazza, Edidin and Kutyniok in Balan et al. (2005) pointed out the following surprising proposition, and proved it detailedly in Balan et al. (2007).

### **Proposition 1**

(Balan et al. 2005,  Theorem 3.2) *Let*$\{f_i\}_{i\in I}$*be a Parseval frame for*${\mathcal {H}}$. *For every subset*$J\subset I$*and every*$f\in {\mathcal {H}}$, *we have*

1 $$\begin{aligned} \sum \limits _{i\in J}|\langle f,\,f_{i}\rangle |^{2}-\left\| \sum \limits _{i\in J}\langle f,\,f_{i}\rangle f_{i}\right\| ^2=\sum \limits _{i\in J^c}|\langle f,\,f_{i}\rangle |^{2}-\left\| \sum \limits _{i\in J^c}\langle f,\,f_{i}\rangle f_{i}\right\| ^2. \end{aligned}$$

Then the study of inequalities related to (1) has interested many mathematicians. The details can be found in Găvruţa (2006); Guo et al. (2016); Li and Sun (2008); Li and Zhu (2012); Zhu and Wu (2010) and references therein. In particular, Balan, Casazza, Edidin and Kutyniok in 2007 and Găvruţa in 2006 obtained following two propositions:

### **Proposition 2**

(Balan et al. 2007,  Proposition 4.1) *Let*$\{f_{j}\}_{j\in J}\subset H$*be a Parseval frame. For any*$f\in H$, $J_{1}\subset J$, *we have*

2 $$\begin{aligned} \sum \limits _{j\in J_{1}}|\langle f,\,f_{j}\rangle |^{2}+\left\| \sum \limits _{j\in J_{1}^{c}}\langle f,\,f_{j}\rangle f_{j} \right\| ^{2}\ge \frac{3}{4}\Vert f\Vert ^{2}. \end{aligned}$$

### **Proposition 3**

(Găvruţa 2006,  Theorem 3.2) *Let*$\{f_{j}\}_{j\in J}\subset H$*be a frame and*$\{g_{j}\}_{j\in J}\subset H$*be an alternate dual frame of*$\{f_{j}\}_{j\in J}$. *then for any*$f\in H$, *we have*

3 $$\begin{aligned} Re\left( \sum \limits _{j\in J_{1}}\langle f,\,g_{j}\rangle \overline{\langle f,\,f_{j}\rangle }\right) +\left\| \sum \limits _{j\in J_{1}^{c}}\langle f,\,g_{j}\rangle f_{j} \right\| ^{2}\ge \frac{3}{4}\Vert f\Vert ^{2}. \end{aligned}$$

Guo, Leng and Li in Guo et al. (2016) generalized Proposition 1 to discrete fusion frames (Guo et al. 2016, Theorem 4). Motivated by above works, in this paper we generalize Proposition 2 and Proposition 3 to continuous fusion frames and fusion pairs. It is worth expecting that our results have potential applications in the frame theory and signal processing. Indeed, our results can be used to recover many results in the literature. For example, Theorem 1 below reduces to Guo et al. (2016), Theorem 8) when the measure is counting measure, and to Proposition 2 if the fusion frame is taken as the usual frame in addition. Similarly, Corollary 3 can be used to recover Proposition 3.

This paper is organized as follows. “Preliminaries” section is an auxiliary one. And in this section, we recall some basic notions and properties. In “Equalities and inequalities for continuous fusion frames” section, using the method of operator theory we obtain some important inequalities for continuous fusion frames which are very different from those in the literature. In “Equalities and inequalities for fusion pairs” section, we derive some inequalities of fusion pairs and some bounds estimates.

## Preliminaries

This section is an auxiliary one. First we recall some basic notations and notions . The readers can refer to Casazza and Kutyniok (2004), Christensen (2003), Faroughi and Ahmadi (2008, 2010), Rahimi et al. (2006) for details.

Let $H,\,K$ be separable Hilbert spaces, and *I* a countable index set. We denote by $I_{H}$ the identity operator on *H*, $\hat{H}$ the collection of all closed subspace of *H*, and $L(H,\,K)$ the set of all bounded linear operators from *H* into *K*. For a positive measure space $(X,\,\mu )$, we always assume that $v:X\rightarrow [0,\,\infty )$ is measurable mapping on *X* satisfying $v(x)\ne 0$ for a.e. $x\in X$.

Let *F* be a mapping from *X* into $\widehat{H}$. We denote by $L^{2}(X,F)$ the set of all measurable mappings $f:X\rightarrow H$ such that, for each $x\in X$, and $f(x)\in F(x)$, and $\int _{X}\Vert f(x)\Vert ^{2}d\mu (x)<\infty$. Then it is a Hilbert space under the following inner product:

$$\begin{aligned} \langle f,g\rangle =\int _{X}\langle f(x),g(x)\rangle d\mu (x) \quad f,g\in L^{2}(X,F). \end{aligned}$$

### **Definition 1**

(Christensen 2003) Let $\{f_{i}:i\in I\}$ be a sequence in *H*, we say that $\{f_{i}:i\in I\}$ is a frame if there exist $0<A_{1}\le B_{1}<+\infty$ such that

$$\begin{aligned} A_{1}\Vert h\Vert ^{2}\le \sum \limits _{i\in I}|\langle f_{i},\,h\rangle |^{2}\le B_{1}\Vert h\Vert ^{2}, \quad \forall h\in H. \end{aligned}$$

The numbers $A_{1}, B_{1}$ are called lower and upper bounds for the frame, respectively.

### **Definition 2**

(Casazza and Kutyniok 2004) Let *H* be a separable Hilbert space, $\{w_{i}:i\in I\}$ be a family of closed subspace of Hilbert space *H*, and $\{v_{i}:i\in I\}$ be a family of weight, i.e., $v_{i}>0$ for all $i\in I$. The family $\{(w_{i},\,v_i):i\in I\}$ is a fusion frame, if there exist constants $0<A_{2}\le B_{2}<+\infty$ such that

$$\begin{aligned} A_{2}\Vert h\Vert ^{2}\le \sum \limits _{i\in I}v_{i}^{2}\Vert \pi _{w_{i}}(h)\Vert ^{2}\le B_{2}\Vert h\Vert ^{2}, \quad \forall h\in H, \end{aligned}$$

where $\pi _{w_{i}}$ is the orthogonal projection onto the subspace $w_{i}$. The numbers $A_{2},B_{2}$ are called lower and upper frame bounds for the fusion frame, respectively.

### **Definition 3**

(Rahimi et al. 2006) Let $(X,\mu )$ be a measure space with positive measure $\mu$, and let $f:X\rightarrow H$ be weakly measurable (i.e., for all $h\in H,$ the mapping $x\rightarrow \langle f(x),h\rangle$ is measurable). Then $\{f(x):x\in X\}$ is called a continuous frame for *H* if there exist constants $0<A_{3}\le B_{3}<+\infty$ such that

4 $$\begin{aligned} A_{3}\Vert h\Vert ^{2}\le \int _{X}|\langle f(x),h\rangle |^{2}d\mu (x)\le B_{3}\Vert h\Vert ^{2}, \quad \forall h\in H. \end{aligned}$$

We call $A_{3}$ and $B_{3}$ the lower and upper continuous frame bounds, respectively. If only the right-hand inequality of (4) is satisfied, we call $\{f(x):x\in X\}$ a continuous Bessel sequence in *H* with Bessel bound $B_{3}$. If $A_{3}=B_{3}=\lambda$ in (4), we call $\{f(x):x\in X\}$ a $\lambda$-tight continuous frame. Moreover, if $\lambda =1$, $\{f(x):x\in X\}$ is called a Parseval continuous frame.

### **Definition 4**

(Faroughi and Ahmadi 2008) Let $F:X\rightarrow \widehat{H}$ be such that for each $h\in H$, the mapping $x\rightarrow \pi _{F_{(x)}}(h)$ is measurable (i.e., *F* is weakly measurable). We say that $(F,\,v)$ is a continuous fusion frame for *H* if there exist constants $0<A\le B<+\infty$ such that

5 $$\begin{aligned} A\Vert h\Vert ^{2}\le \int _{X}v^{2}(x)\Vert \pi _{F(x)}(h)\Vert ^{2}d\mu (x)\le B\Vert h\Vert ^{2}, \quad \forall h\in H, \end{aligned}$$

where $\pi _{F(x)}$ is the orthogonal projection onto the space *F*(*x*). The numbers *A*, *B* are called lower and upper frame bounds for the continuous fusion frame, respectively. If only the right-hand inequality of (5) is satisfied, we call $(F,\,v)$ a continuous Bessel fusion mapping on *H* with bound *B*. If $A=B=\lambda$ in (5), we call $(F,\,v)$ a $\lambda$-tight continuous fusion frame. Moreover, if $\lambda =1$, $(F,\,v)$ is called a Parsevel continuous fusion frame.

### *Remark 1*

A continuous fusion frame is a generalization of fusion frame. Indeed, when *X* is countable, and $\mu$ is a counting measure, it is exactly a fusion frame.

Let $(F,\,v)$ be a continuous fusion frame for *H*. In Faroughi and Ahmadi (2008), the authors defined the continuous fusion frame operator $S_{F}: H\rightarrow H$ as follows:

$$\begin{aligned} S_{F}(h)=\int _{X}v^{2}(x)\pi _{F(x)}(h)d\mu (x), \quad \forall h\in H. \end{aligned}$$

It is easy to show that $S_{F}$ is a bounded, positive, self-adjoint and invertible operator.

For any $X_{1}\subset X$, denote $X_{1}^{c}=X\backslash X_{1}$, and we define the following operators:

$$\begin{aligned} S_{F}^{X_{1}}h &=   \int _{X_{1}}v^{2}(x)\pi _{F(x)}(h)d\mu (x), \quad \forall h\in H. \\ S_{F}^{X_{1}^{c}}h&=   \int _{X_{1}^{c}}v^{2}(x)\pi _{F(x)}(h)d\mu (x), \quad\forall h\in H. \end{aligned}$$

Then $S_{F}=S_{F}^{X_{1}}+S_{F}^{X_{1}^{c}}$, and $S_{F}^{X_{1}},\,S_{F}^{X_{1}^{c}}$ are positive and self-adjoin operators.

### **Definition 5**

(Faroughi and Ahmadi 2010) Let $(F,\,v)$ and $(G,\,v)$ be continuous Bessel fusion mappings on *H*. We say that *F* and *G* is a fusion pair if for any $h\in H$ the following holds

$$\begin{aligned} h=\int _{X}v^{2}(x)\pi _{G(x)}\pi _{F(x)}(h)d\mu (x) =\int _{X}v^{2}(x)\pi _{F(x)}\pi _{G(x)}(h)d\mu (x). \end{aligned}$$

## Equalities and inequalities for continuous fusion frames

This section is devoted to some inequalities for continuous fusion frames. For this purpose, we first give a simple property of self-adjoint operators.

### **Lemma 1**

*Let*$T\in L(H)$*be a self-adjoint operator and*$a,\,b,\,c\in {\mathcal {R}},\,U=aT^{2}+bT+cI_{H}$, *then the following statements hold*.

(i) *if*$$a>0$$, *then*
  $$\begin{aligned} U\ge \frac{4ac-b^{2}}{4a}I_{H}. \end{aligned}$$
(ii) *if*$a<0$, *then*
  $$\begin{aligned} U\le \frac{4ac-b^{2}}{4a}I_{H}. \end{aligned}$$

### *Proof*

We only prove (i), and (ii) can be proved similarly. It is easy to check that

$$\begin{aligned} U=a\left(T+\frac{b}{2a}I_{H}\right)^{2}+\frac{4ac-b^{2}}{4a}I_{H}. \end{aligned}$$

Observing that $(T+\frac{b}{2a}I_{H})^{2}$ is a positive operator, we have (i). $\square$

### **Lemma 2**

(Găvruţa 2006, Theorem 2.1) *If*$T_{1},\,T_{2}\in L(H)$*are bounded, self-adjoint linear operator satisfying*$T_{1}+T_{2}=I_{H}$, *then for all*$h\in H$, *we have*

$$\begin{aligned} \langle T_{1}h,h\rangle +\Vert T_{2}h\Vert ^{2}=\langle T_{2}h,h\rangle +\Vert T_{1}h\Vert ^{2}\ge \frac{3}{4}\Vert h\Vert ^{2}. \end{aligned}$$

### **Theorem 1**

*Let* (*F*, *v*) *be a Parseval continuous fusion frame forH*. *Then for*$X_{1}\subset X$*and*$h\in H$, *we have*

6 $$\begin{aligned} 0\le&\int _{X_{1}}v^{2}(x)\Vert \pi _{F(x)}(h)\Vert ^{2}d\mu (x)-\left\| \int _{X_{1}}v^{2}(x)\pi _{F(x)}(h)d\mu (x)\right\| ^{2} \le \frac{1}{4}\Vert h\Vert ^{2}, \end{aligned}$$

7 $$\begin{aligned} \frac{1}{2}\Vert h\Vert ^{2}\le \left\| \int _{X_{1}}v^{2}(x)\pi _{{F}(x)}(h)d\mu (x)\right\| ^{2}+ \left\| \int _{X_{1}^{c}}v^{2}(x)\pi _{{F}(x)}(h)d\mu (x)\right\| ^{2}\le \frac{3}{2}\Vert h\Vert ^{2}, \end{aligned}$$

8 $$\begin{aligned} \frac{3}{4}\Vert h\Vert ^{2}\le&\int _{X_{1}}v^{2}(x)\Vert \pi _{F(x)}(h)\Vert ^{2}d\mu (x)+\left\| \int _{X_{1}^{c}}v^{2}(x)\pi _{F(x)}(h)d\mu (x)\right\| ^{2} \le \Vert h\Vert ^{2}. \end{aligned}$$

### *Proof*

Suppose that $(F,\,v)$ is a Parseval continuous fusion frame for *H*, then $S_{F}$ is invertible and positive on *H* and $S_{F}^{X_{1}}+S_{F}^{X_{1}^{c}}=I_{H}$. By a simple calculation, we have $S_{F}^{X_{1}}S_{F}^{X_{1}^{c}}=S_{F}^{X_{1}^{c}}S_{F}^{X_{1}}$. It follows that

9 $$\begin{aligned} 0\le S_{F}^{X_{1}}S_{F}^{X_{1}^{c}}=S_{F}^{X_{1}}(I_{H}-S_{F}^{X_{1}})=S_{F}^{X_{1}}-(S_{F}^{X_{1}})^{2}, \end{aligned}$$

and thus

10 $$ S_{F}^{X_{1}}-(S_{F}^{X_{1}})^{2}\le \frac{1}{4}I_{H}. $$

by Lemma 1. For $h\in H$, we have

11 $$\begin{aligned} \langle (S_{F}^{X_{1}}-(S_{F}^{X_{1}})^{2})h,\,h\rangle &=\langle S_{F}^{X_{1}}h,\,h\rangle -\langle (S_{F}^{X_{1}})^{2}h,\,h\rangle \nonumber \\ &=\int _{X_{1}}v^{2}(x)\Vert \pi _{F(x)}(h)\Vert ^{2}d\mu (x)-\left\| \int _{X_{1}}v^{2}(x)\pi _{F(x)}(h)d\mu (x)\right\| ^{2}. \end{aligned}$$

Combining (9) and (10), we get (6).

Observe that

12 $$\begin{aligned} (S_{F}^{X_{1}})^{2}+(S_{F}^{X_{1}^{c}})^{2}&=(S_{F}^{X_{1}})^{2}+(I_{H}-S_{F}^{X_{1}})^{2} \nonumber \\ &=2(S_{F}^{X_{1}})^{2}-2S_{F}^{X_{1}}+I_{H}. \end{aligned}$$

It follows that

13 $$\begin{aligned} (S_{F}^{X_{1}})^{2}+(S_{F}^{X_{1}^{c}})^{2}\ge \frac{1}{2}I_{H}. \end{aligned}$$

by Lemma 1. Also observing that $S_{F}^{X_{1}}-(S_{F}^{X_{1}})^{2}\ge 0$ and

14 $$\begin{aligned} (S_{F}^{X_{1}})^{2}+(S_{F}^{X_{1}^{c}})^{2}&=2(S_{F}^{X_{1}})^{2}-2S_{F}^{X_{1}}+I_{H} \nonumber \\ &=I_{H}+2S_{F}^{X_{1}}-2(S_{F}^{X_{1}})^{2}+4((S_{F}^{X_{1}})^{2}-S_{F}^{X_{1}}), \end{aligned}$$

we have

$$\begin{aligned} (S_{F}^{X_{1}})^{2}+(S_{F}^{X_{1}^{c}})^{2}\le I_{H}+2S_{F}^{X_{1}}-2(S_{F}^{X_{1}})^{2}. \end{aligned}$$

Again by Lemma 1, we get

15 $$\begin{aligned} (S_{F}^{X_{1}})^{2}+(S_{F}^{X_{1}^{c}})^{2}\le \frac{3}{2}I_{H} \end{aligned}$$

Since

16 $$\begin{aligned} \langle (S_{F}^{X_{1}})^{2}+(S_{F}^{X_{1}^{c}})^{2}h,\,h\rangle &=\langle S_{F}^{X_{1}}h,\,S_{F}^{X_{1}}h \rangle +\langle S_{F}^{X_{1}^{c}}h,\,S_{F}^{X_{1}^{c}}h \rangle \nonumber \\ &=\left\| \int _{X_{1}}v^{2}(x)\pi _{{F}(x)}(h)d\mu (x)\right\| ^{2}+ \left\| \int _{X_{1}^{c}}v^{2}(x)\pi _{{F}(x)}(h)d\mu (x)\right\| ^{2} \end{aligned}$$

for $h\in H$, we have (7) by (13) and (15).

Next we prove (8). Observe that

17 $$\begin{aligned} S_{F}^{X_{1}}+(S_{F}^{X_{1}^{c}})^{2}&=S_{F}^{X_{1}}+(I_{H}-S_{F}^{X_{1}})^{2} \nonumber \\ &=(S_{F}^{X_{1}})^{2}-S_{F}^{X_{1}}+I_{H}, \end{aligned}$$

and that $S_{F}^{X_{1}}-(S_{F}^{X_{1}})^{2}\ge 0$ by (9). It leads to

18 $$\begin{aligned} \frac{3}{4}I_{H}\le S_{F}^{X_{1}}+(S_{F}^{X_{1}^{c}})^{2}\le I_{H} \end{aligned}$$

by Lemma 1. For $h\in H$, we have

19 $$\begin{aligned} \langle S_{F}^{X_{1}}+(S_{F}^{X_{1}^{c}})^{2}h,\,h\rangle &=\langle S_{F}^{X_{1}}h,\,h \rangle +\langle S_{F}^{X_{1}^{c}}h,\,S_{F}^{X_{1}^{c}}h \rangle \nonumber \\ &=\int _{X_{1}}v^{2}(x)\Vert \pi _{{F}(x)}(h)\Vert ^{2}d\mu (x)+ \left\| \int _{X_{1}^{c}}v^{2}(x)\pi _{{F}(x)}(h)d\mu (x)\right\| ^{2}. \end{aligned}$$

Collecting (18) and (19) leads to (8). The proof is completed. $\square$

Observe that $(\frac{1}{\sqrt{\lambda }}F,v)$ is a Parseval continuous fusion frame if (*F*, *v*) is a $\lambda$-tight continuous fusion frame for *H*. As an immediate consequence of Theorem 3.1, we have

### **Corollary 1**

*Let* (*F*, *v*) *be a*$\lambda$-*tight continuous fusion frame forH*. *Then for*$X_{1}\subset X$*and*$h\in H$, *we have*

$$\begin{aligned} 0&\le   \lambda \int _{X_{1}}v^{2}(x)\Vert \pi _{F(x)}(h)\Vert ^{2}d\mu (x)- \left\| \int _{X_{1}}v^{2}(x)\pi _{F(x)}(h)d\mu (x)\right\| ^{2}\le \frac{\lambda ^{2}}{4}\Vert h\Vert ^{2}\\ \frac{\lambda ^{2}}{2}\Vert h\Vert ^{2}&\le   \left\| \int _{X_{1}}v^{2}(x)\pi _{{F}(x)}(h)d\mu (x)\right\| ^{2}+ \left\| \int _{X_{1}^{c}}v^{2}(x)\pi _{{F}(x)}(h)d\mu (x)\right\| ^{2}\le \frac{3\lambda ^{2}}{2}\Vert h\Vert ^{2}\\ \frac{3\lambda ^{2}}{4}\Vert h\Vert ^{2}&\le  \lambda \int _{X_{1}}v^{2}(x)\Vert \pi _{F(x)}(h)\Vert ^{2}d\mu (x)+\left\| \int _{X_{1}^{c}}v^{2}(x)\pi _{F(x)}(h)d\mu (x)\right\| ^{2} \le \lambda ^{2}\Vert h\Vert ^{2}. \end{aligned}$$

Next we will give a equality for tight continuous fusion frames. To do so, we first define two operators $S_{F}^{1}$, $S_{F}^{2}$ as follows:

20 $$\begin{aligned} S_{F}^{1}: H\rightarrow H, \quad S_{F}^{1}(h)=&\int _{X}a_{x}v^{2}(x)\pi _{F(x)}(h)d\mu (x), \quad \forall h\in H, \end{aligned}$$

21 $$\begin{aligned} S_{F}^{2}: H\rightarrow H, \quad S_{F}^{2}(h)=&\int _{X}(1-a_{x})v^{2}(x)\pi _{F(x)}(h)d\mu (x), \quad \forall h\in H, \end{aligned}$$

where $(F,\,v)$ is a continuous Bessel fusion mapping on *H* and $\{a_{x}:x\in X\}\in l^{\infty }(X)$, $l^{\infty }(X)=\left\{ \{a_{x}:x\in X\}:\sup \nolimits _{x\in X}|a_{x}|<\infty \right\}$.

### **Proposition 4**

*Let*$(F,\,v)$*be a continuous Bessel fusion mapping onHwith boundB*, *then*$S_{F}^{1}$, $S_{F}^{2}$*are bounded linear operators, and*

$$\begin{aligned} (S_{F}^{1})^{*}(h)= & {} \int _{X}\bar{a}_{x}v^{2}(x)\pi _{F(x)}(h)d\mu (x),\quad \forall h\in H.\\ (S_{F}^{2})^{*}(h)= & {} \int _{X}(1-\bar{a}_{x})v^{2}(x)\pi _{F(x)}(h)d\mu (x),\quad \forall h\in H. \end{aligned}$$

### *Proof*

We only treat $S_{F}^{1}$, and the other part $S_{F}^{2}$ can be treated similarly. For $h\in H$ and $X_{1}\subset X$, we have

$$\begin{aligned} \left\| \int _{X_{1}}a_{x}v^{2}(x)\pi _{{F}(x)}(h)d\mu (x)\right\| &=\sup \limits _{g\in H,\Vert g\Vert =1}\left| \left\langle \int _{X_{1}}a_{x}v^{2}(x)\pi _{{F}(x)}(h)d\mu (x),\,g\right\rangle \right| \\ &=\sup \limits _{g\in H,\Vert g\Vert =1}\left| \int _{X_{1}}v^{2}(x)\left\langle \pi _{{F}(x)}h,\,\bar{a}_{x}g\right\rangle d\mu (x)\right| \\ &=\sup \limits _{g\in H,\Vert g\Vert =1}\left| \int _{X_{1}}v^{2}(x)\left\langle \pi _{{F}(x)}h,\,\bar{a}_{x}\pi _{{F}(x)}g\right\rangle d\mu (x)\right| \\ &\le  \sup \limits _{g\in H,\Vert g\Vert =1}\left( \int _{X_{1}}v^{2}(x)\Vert \pi _{{F}(x)}(h)\Vert ^{2}d\mu (x)\right) ^{\frac{1}{2}}\\ &\quad \times \left( \int _{X_{1}}v^{2}(x)\Vert \bar{a}_{x}\pi _{{F}(x)}(h)\Vert ^{2}d\mu (x)\right) ^{\frac{1}{2}}\\ &\le BM\Vert h\Vert , \end{aligned}$$

where $M=\sup \nolimits _{x\in X}|a_{x}|$ and $\overline{a}_{x}$ is the conjugate of $a_{x}$. This implies that $S_{F}^{1}$ is well-defined and $\Vert S_{F}^{1}h\Vert \le BM\Vert h\Vert$. Therefore, $S_{F}^{1}$ is a bounded linear operator. Now let us compute $(S_{F}^{1})^{*}$,

$$\begin{aligned} \langle h,\,(S_{F}^{1})^{*}(f)\rangle =\langle S_{F}^{1}h,\,f\rangle &=\left\langle \int _{X}a_{x}v^{2}(x)\pi _{F(x)}(h)d\mu (x),\,f\right\rangle \\ &=\int _{X}v^{2}(x) \langle \pi _{F(x)}(h),\,\bar{a}_{x}f\rangle d\mu (x)\\ &=\int _{X}v^{2}(x) \langle h,\,\bar{a}_{x}\pi _{F(x)}(f)\rangle d\mu (x)\\ &=\left\langle h,\,\int _{X}\bar{a}_{x}v^{2}(x)\pi _{F(x)}(f)d\mu (x)\right\rangle . \end{aligned}$$

The proof is completed. $\square$

### **Theorem 2**

*Let*$(F,\,v)$*be a*$\lambda$-*tight continuous fusion frame forH*. *Then for*$h\in H$*and*$\{a_{x}:x\in X\}\in l^{\infty }(X)$, *we have*

$$\begin{aligned}&\lambda \int _{X}a_{x}v^{2}(x)\Vert \pi _{F(x)}(h)\Vert ^{2}d\mu (x)+\left\| \int _{X}(1-a_{x})v^{2}(x)\pi _{F(x)}(h)d\mu (x)\right\| ^{2}\\&\quad =\lambda \int _{X}(1-\bar{a}_{x})v^{2}(x)\Vert \pi _{F(x)}(h)\Vert ^{2}d\mu (x)+\left\| \int _{X}a_{x}v^{2}(x)\pi _{{F}(x)}(h)d\mu (x)\right\| ^{2}, \end{aligned}$$

*where*$\overline{a}_{x}$*is the conjugate of*$a_{x}$.

### *Proof*

By Proposition 4, $S_{F}^{1},\,S_{F}^{2}$ are well-defined and

$$\begin{aligned} S_{F}^{1}h+S_{F}^{2}h=\int _{X}v^{2}(x)\pi _{F(x)}(h)d\mu (x)=S_{F}h, \quad \forall h\in H. \end{aligned}$$

Since $(F,\,v)$ is a $\lambda$-tight continuous fusion frame for *H*, that is $\lambda ^{-1}S_{F}^{1}+\lambda ^{-1}S_{F}^{2}=I_{H}$. Write $Q_{1}=\lambda ^{-1}S_{F}^{1}$ and $Q_{2}=\lambda ^{-1}S_{F}^{2}$, then

$$\begin{aligned} Q_{1}+Q_{2}^{*}Q_{2}&=Q_{1}+(I_{H}-Q_{1})^{*}(I_{H}-Q_{1})\\ &=Q_{1}+(I_{H}-Q_{1}^{*})(I_{H}-Q_{1})\\&=Q_{1}+I_{H}-Q_{1}-Q_{1}^{*}+Q_{1}^{*}Q_{1}\\ &=I_{H}-Q_{1}^{*}+Q_{1}^{*}Q_{1}\\ &=Q_{2}^{*}+Q_{1}^{*}Q_{1}, \end{aligned}$$

and thus

$$\begin{aligned} \lambda S_{F}^{1}+(S_{F}^{2})^{*}S_{F}^{2}=\lambda S_{F}^{2}+(S_{F}^{1})^{*}S_{F}^{1}. \end{aligned}$$

Hence for $h\in H$, we get

$$\begin{aligned}&\lambda \int _{X}a_{x}v^{2}(x)\Vert \pi _{F(x)}(h)\Vert ^{2}d\mu (x)+\left\| \int _{X}(1-a_{x})v^{2}(x)\pi _{F(x)}(h)d\mu (x)\right\| ^{2}\\&\quad =\langle \lambda S_{F}^{1}h,h\rangle +\langle (S_{F}^{2})^{*}S_{F}^{2}h,h\rangle \\&\quad =\langle (\lambda S_{F}^{1}+(S_{F}^{2})^{*}S_{F}^{2})h,h\rangle \\&\quad =\langle (\lambda S_{F}^{2}+(S_{F}^{1})^{*}S_{F}^{1})h,h\rangle \\&\quad =\langle \lambda (S_{F}^{2})^{*}h,h\rangle +\langle (S_{F}^{1})^{*}S_{F}^{1}h,h\rangle \\&\quad =\langle h,S_{F}^{2}h\rangle +\Vert S_{F}^{1}h\Vert ^{2}\\&\quad =\lambda \int _{X}(1-\overline{a}_{x})v^{2}(x)\Vert \pi _{F(x)}(h)\Vert ^{2}d\mu (x)+\left\| \int _{X}a_{x}v^{2}(x)\pi _{{F}(x)}(h)d\mu (x)\right\| ^{2}. \end{aligned}$$

The proof is completed. $\square$

As an immediate consequence of Theorem 2 and Lemma 2, we have

### **Corollary 2**

*Let*$(F,\,v)$*be a*$\lambda$-*tight continuous fusion frame forH*, $\{a_{x}:x\in X\}\in l^{\infty }(X)$*with*$a_{x}$*being real. Then for*$h\in H$, *we have*

$$\begin{aligned}&\lambda \int _{X}a_{x}v^{2}(x)\Vert \pi _{F(x)}(h)\Vert ^{2}d\mu (x)+\left\| \int _{X}(1-a_{x})v^{2}(x)\pi _{F(x)}(h)d\mu (x)\right\| ^{2}\\&\quad =\lambda \int _{X}(1-a_{x})v^{2}(x)\Vert \pi _{F(x)}(h)\Vert ^{2}d\mu (x)+\left\| \int _{X}a_{x}v^{2}(x)\pi _{{F}(x)}(h)d\mu (x)\right\| ^{2}\ge \frac{3}{4}\Vert h\Vert ^{2}. \end{aligned}$$

## Equalities and inequalities for fusion pairs

This section focuses on fusion pairs. We begin with the following lemma which can be proved similarly to Proposition 4.

### **Lemma 3**

*Let*$(F,\,v)$*and*$(G,\,v)$*be continuous Bessel fusion mappings onH*, *FandGbe a fusion pair, and*$\{a_{x}:x\in X\}\in l^{\infty }(X)$. *Define the operator*$T_{a}$*as follows*:

$$\begin{aligned} T_{a}: H\rightarrow H, T_{a}h=\int _{X}a_{x}v^{2}(x)\pi _{F(x)}\pi _{G(x)}(h)d\mu (x),\quad \forall h\in H; \end{aligned}$$

*then*$T_{a}$*is a bounded linear operator, and*

$$\begin{aligned} T_{a}^{*}h=\int _{X}\bar{a}_{x}v^{2}(x)\pi _{G(x)}\pi _{F(x)}(h)d\mu (x) \end{aligned}$$

### **Theorem 3**

*Let*$(F,\,v)$*and*$(G,\,v)$*be continuous Bessel fusion mappings onH*, *FandGbe a fusion pair, and*$\{a_{x}:x\in X\}\in l^{\infty }(X)$. *Then for*$h\in H$, *we have*

22 $$\begin{aligned}&\left\| \int _{X}a_{x}v^{2}(x)\pi _{F(x)}\pi _{G(x)}(h)d\mu (x)\right\| ^{2}+\int _{X}(1-a_{x})v^{2}(x)\langle \pi _{G(x)}(h),\,\pi _{F(x)}(h)\rangle d\mu (x)\nonumber \\&\quad =\left\| \int _{X}(1-a_{x})v^{2}(x)\pi _{F(x)}\pi _{G(x)}(h)d\mu (x)\right\| ^{2}+\overline{\int _{X}a_{x}v^{2}(x) \langle \pi _{G(x)}(h),\,\pi _{F(x)}(h)\rangle d\mu (x)} \end{aligned}$$

### *Proof*

Let $$T_{1-a}h=\int _{X}(1-a_{x})v^{2}(x)\pi _{F(x)}\pi _{G(x)}(h)d\mu (x), \forall h\in H$$, then

$$\begin{aligned} T_{1-a}^{*}h=\int _{X}(1-\bar{a}_{x})v^{2}(x)\pi _{G(x)}\pi _{F(x)}(h)d\mu (x),\quad \forall h\in H, \end{aligned}$$

and $T_{a}+T_{1-a}=I_{H}$. So for $h\in H$, we have

23 $$\begin{aligned}&\left\| \int _{X}a_{x}v^{2}(x)\pi _{F(x)}\pi _{G(x)}(h)d\mu (x)\right\| ^{2}+\int _{X}(1-a_{x})v^{2}(x)\langle \pi _{G(x)}(h),\,\pi _{F(x)}(h)\rangle d\mu (x)\nonumber \\&\quad =\Vert T_{a}h\Vert ^{2}+\langle T_{1-a}h,\,h \rangle =\langle T_{a}h,\,T_{a}h\rangle +\langle (I_{H}- T_{a})h,\,h \rangle \nonumber \\&\quad = \langle T_{a}h,\,T_{a}h\rangle +\langle h,\,h\rangle -\langle T_{a}h,\,h\rangle . \end{aligned}$$

On the other hand,

24 $$\begin{aligned}&\left\| \int _{X}(1-a_{x})v^{2}(x)\pi _{F(x)}\pi _{G(x)}(h)d\mu (x)\right\| ^{2}+\overline{\int _{X}a_{x}v^{2}(x) \langle \pi _{G(x)}(h),\,\pi _{F(x)}(h)\rangle d\mu (x)}\nonumber \\&\quad =\Vert T_{1-a}h\Vert ^{2}+\overline{\langle T_{a}h,\,h \rangle }= \langle T_{1-a}h,\,T_{1-a}h\rangle +\langle h,\,T_{a}h \rangle \nonumber \\&\quad = \langle (I_{H}- T_{a})h,\,(I_{H}- T_{a})h\rangle +\langle h,\,T_{a}h \rangle \nonumber \\&\quad =\langle h,\,h\rangle -\langle T_{a}h,\,h\rangle +\langle T_{a}h,\,T_{a}h\rangle . \end{aligned}$$

Therefore, (22) holds by (23) and (24). The proof is completed. $\square$

### *Remark 2*

Theorem 2 is a special case of Theorem 3.

### **Theorem 4**

*Let*$(F,\,v)$*and*$(G,\,v)$*be continuous Bessel fusion mappings onH*, *FandGbe a fusion pair, and*$\{a_{x}:x\in X\}\in l^{\infty }(X)$. *Then for*$h\in H$, *we have*

25 $$\begin{aligned}&\left\| \int _{X}a_{x}v^{2}(x)\pi _{F(x)}\pi _{G(x)}(h)d\mu (x)\right\| ^{2}+Re\left(\int _{X}(1-a_{x})v^{2}(x)\langle \pi _{G(x)}(h),\,\pi _{F(x)}(h)\rangle d\mu (x)\right)\nonumber \\&\quad =\left\| \int _{X}(1-a_{x})v^{2}(x)\pi _{F(x)}\pi _{G(x)}(h)d\mu (x)\right\| ^{2}+Re\left( \int _{X}a_{x}v^{2}(x) \langle \pi _{G(x)}(h),\,\pi _{F(x)}(h)\rangle d\mu (x)\right) \ge \frac{3}{4}\Vert h\Vert ^{2} \end{aligned}$$

### *Proof*

By Theorem 3, for $h\in H$, we have

$$\begin{aligned}&\left\| \int _{X}a_{x}v^{2}(x)\pi _{F(x)}\pi _{G(x)}(h)d\mu (x)\right\| ^{2}+Re\left(\int _{X}(1-a_{x})v^{2}(x)\langle \pi _{G(x)}(h),\,\pi _{F(x)}(h)\rangle d\mu (x)\right)\\&\quad =\left\| \int _{X}(1-a_{x})v^{2}(x)\pi _{G(x)}\pi _{F(x)}(h)d\mu (x)\right\| ^{2}+Re\left(\int _{X}a_{x}v^{2}(x) \langle \pi _{F(x)}(h),\,\pi _{G(x)}(h)\rangle d\mu (x)\right).\end{aligned}$$

Next we prove the “inequality” part. By Lemma 3, we have

$$\begin{aligned} T_{a}h=\int _{X}a_{x}v^{2}(x)\pi _{F(x)}\pi _{G(x)}(h)d\mu (x),\quad \forall h\in H, \end{aligned}$$

and

$$\begin{aligned} T_{a}^{*}h=\int _{X}\bar{a}_{x}v^{2}(x)\pi _{G(x)}\pi _{F(x)}(h)d\mu (x),\quad \forall h\in H. \end{aligned}$$

It follows that

$$\begin{aligned} Re\left( \int _{X}a_{x}v^{2}(x) \langle \pi _{G(x)}(h),\,\pi _{F(x)}(h)\rangle d\mu (x)\right) =\left\langle \frac{T_{a}+T_{a}^{*}}{2}h,\,h\right\rangle . \end{aligned}$$

Thus for $h\in H$, we have

$$\begin{aligned}&\left\| \int _{X}(1-a_{x})v^{2}(x)\pi _{F(x)}\pi _{G(x)}(h)d\mu (x)\right\| ^{2} +Re\left( \int _{X}a_{x}v^{2}(x) \langle \pi _{G(x)}(h),\,\pi _{F(x)}(h)\rangle d\mu (x)\right) \\&\quad =\left\langle \left( T_{1-a}^{*}T_{1-a}+\frac{T_{a}+T_{a}^{*}}{2}\right) h,\,h\right\rangle \\&\quad =\left\langle \left( (I_{H}-T_{a}^{*})(I_{H}-T_{a})+\frac{T_{a}+T_{a}^{*}}{2}\right) h,\,h\right\rangle \\&\quad =\left\langle \left( I_{H}+T_{a}^{*}T_{a}-\frac{T_{a}+T_{a}^{*}}{2}\right) h,\,h\right\rangle \\&\quad =\left\langle \left[ \left( T_{a}-\frac{1}{2}I_{H}\right) ^{*}\left( T_{a}-\frac{1}{2}I_{H}\right) +\frac{3}{4}I\right] h,\,h\right\rangle \\&\quad =\left\| \left( T_{a}-\frac{1}{2}I_{H}\right) h\right\| ^{2}+\frac{3}{4}\Vert h\Vert ^{2}\ge \frac{3}{4}\Vert h\Vert ^{2}. \end{aligned}$$

The proof is completed. $\square$

### *Remark 3*

Corollary 2 is a special case of Theorem 4.

Take

$$\begin{aligned} a_{x}= \left\{ \begin{aligned} 1,\quad x\in X_{1};\\ 0,\quad x\in X_{1}^{c} \end{aligned}\right. \end{aligned}$$

in Theorem 3, where $X_{1}\subset X$. Then we have

### **Corollary 3**

*Let*$X_{1}\subset X$, $(F,\,v)$*and*$(G,\,v)$*be continuous Bessel fusion mappings onH*, *FandGbe a fusion pair. Then for*$h\in H$, *we have*

26 $$\begin{aligned}&\left\| \int _{X_{1}}v^{2}(x)\pi _{F(x)}\pi _{G(x)}(h)d\mu (x)\right\| ^{2}+Re\left( \int _{X_{1}^{c}}v^{2}(x)\langle \pi _{G(x)}(h),\,\pi _{F(x)}(h)\rangle d\mu (x)\right) \nonumber \\&\quad =\left\| \int _{X_{1}^{c}}v^{2}(x)\pi _{F(x)}\pi _{G(x)}(h)d\mu (x)\right\| ^{2}+Re\left( \int _{X_{1}}v^{2}(x) \langle \pi _{G(x)}(h),\,\pi _{F(x)}(h)\rangle d\mu (x)\right) \ge \frac{3}{4}\Vert h\Vert ^{2} \end{aligned}$$

### **Theorem 5**

*Let*$(F,\,v)$*and*$(G,\,v)$*be continuous Bessel fusion mappings onH*, *FandGbe a fusion pair, and*$\{a_{x}:x\in X\}\in l^{\infty }(X)$. *Then for*$h\in H$, *we have*

27 $$\begin{aligned} \frac{3}{4}\Vert h\Vert ^{2}&\le \left\| \int _{X}a_{x}v^{2}(x)\pi _{F(x)}\pi _{G(x)}(h)d\mu (x)\right\| ^{2} +Re\left( \int _{X}(1-a_{x})v^{2}(x)\langle \pi _{G(x)}(h),\,\pi _{F(x)}(h)\rangle d\mu (x)\right) \nonumber \\&=\left\| \int _{X}(1-a_{x})v^{2}(x)\pi _{F(x)}\pi _{G(x)}(h)d\mu (x)\right\| ^{2}+Re\left( \int _{X}a_{x}v^{2}(x) \langle \pi _{G(x)}(h),\,\pi _{F(x)}(h)\rangle d\mu (x)\right) \nonumber \\&\le \frac{3+\Vert T_{a}-T_{1-a}\Vert ^{2}}{4}\Vert h\Vert ^{2} \end{aligned}$$

### *Proof*

By Theorem 4, the left-hand inequality of (27) holds. Next we prove the right-hand inequality. Observe that $T_{a}+T_{1-a}=I_{H}$. For $h\in H$, we have

$$\begin{aligned}&\left\| \int _{X}(1-a_{x})v^{2}(x)\pi _{F(x)}\pi _{G(x)}(h)d\mu (x)\right\| ^{2} +Re\left( \int _{X}a_{x}v^{2}(x) \langle \pi _{G(x)}(h),\,\pi _{F(x)}(h)\rangle d\mu (x)\right) \\&\quad =\left\langle T_{1-a}h,\,T_{1-a}h\right\rangle +Re\left\langle T_{a}h,\,h\right\rangle \\&\quad = \left\langle T_{1-a}h,\,T_{1-a}h\right\rangle +\langle h,\,h\rangle -Re\left\langle T_{1-a}h,\,h\right\rangle \\&\quad = \frac{3}{4}\langle h,\,h\rangle +\frac{1}{4}\langle h,\,h\rangle -Re\left\langle T_{1-a}h,\,h\right\rangle +\left\langle T_{1-a}h,\,T_{1-a}h\right\rangle \\&\quad = \frac{3}{4}\langle h,\,h\rangle +\frac{1}{4}\left( \langle h,\,h\rangle -4Re\left\langle T_{1-a}h,\,h\right\rangle +4\left\langle T_{1-a}h,\,T_{1-a}h\right\rangle \right) \\&\quad =\frac{3}{4}\langle h,\,h\rangle +\frac{1}{4}\left( \langle h,\,h\rangle -2\left\langle T_{1-a}h,\,h\right\rangle -2\left\langle h,\,T_{1-a}h\right\rangle +4\left\langle T_{1-a}h,\,T_{1-a}h\right\rangle \right) \\&\quad =\frac{3}{4}\langle h,\,h\rangle +\frac{1}{4}\left\langle (I_{H}-2T_{1-a})h,\,(I_{H}-2T_{1-a})h\right\rangle \\&\quad = \frac{3}{4}\langle h,\,h\rangle +\frac{1}{4}\left\langle (T_{a}-T_{1-a})h,\,(T_{a}-T_{1-a})h\right\rangle \\&\quad \le \frac{3}{4}\Vert h\Vert ^{2}+\frac{1}{4}\Vert T_{a}-T_{1-a}\Vert ^{2}\Vert h\Vert ^{2}\\&\quad =\frac{3+\Vert T_{a}-T_{1-a}\Vert ^{2}}{4}\Vert h\Vert ^{2}. \end{aligned}$$

The proof is completed. $\square$

Let $X_{1}\subset X$, $(F,\,v)$ and $(G,\,v)$ be continuous Bessel fusion mappings on *H*, *F* and *G* be a fusion pair. Define the operators $L_{X_{1}},\,L_{X_{1}^{c}}$ as follows:

$$\begin{aligned} L_{X_{1}}: H\rightarrow H, L_{X_{1}}h= & {} \int _{X_{1}}v^{2}(x)\pi _{F(x)}\pi _{G(x)}(h)d\mu (x),\quad \forall h\in H;\\ L_{X_{1}^{c}}: H\rightarrow H, L_{X_{1}^{c}}h= & {} \int _{X_{1}^{c}}v^{2}(x)\pi _{F(x)}\pi _{G(x)}(h)d\mu (x),\quad \forall h\in H. \end{aligned}$$

It is easy to prove $L_{X_{1}},\,L_{X_{1}^{c}}$ are bounded linear operators. As an immediate consequence of Theorem 5 and Corollary 3, we have

### **Corollary 4**

*Let*$X_{1}\subset X$, $(F,\,v)$*and*$(G,\,v)$*be continuous Bessel fusion mappings forH*, *FandGbe a fusion pair. Then for*$h\in H$, *we have*

28 $$\begin{aligned} \frac{3}{4}\Vert h\Vert ^{2}&\le \left\| \int _{X_{1}}v^{2}(x)\pi _{F(x)}\pi _{G(x)}(h)d\mu (x)\right\| ^{2} +Re\left( \int _{X_{1}^{c}}v^{2}(x)\langle \pi _{G(x)}(h),\,\pi _{F(x)}(h)\rangle d\mu (x)\right) \nonumber \\&=\left\| \int _{X_{1}^{c}}v^{2}(x)\pi _{F(x)}\pi _{G(x)}(h)d\mu (x)\right\| ^{2}+Re\left( \int _{X_{1}}v^{2}(x) \langle \pi _{G(x)}(h),\,\pi _{F(x)}(h)\rangle d\mu (x)\right) \nonumber \\&\le \frac{3+\Vert L_{X_{1}}-L_{X_{1}^{c}}\Vert ^{2}}{4}\Vert h\Vert ^{2} \end{aligned}$$

## Conclusions

In this paper, we obtain three inequalities for Parseval continuous fusion frames, an equality for $\lambda$-tight continuous fusion frames and an inequality for fusion pairs (see Theorems 3.1, 3.2 and 4.3). These results can recover some well known frames inequalities.
